# The fitness for the Ageing Brain Study II (FABS II): protocol for a randomized controlled clinical trial evaluating the effect of physical activity on cognitive function in patients with Alzheimer's disease

**DOI:** 10.1186/1745-6215-11-120

**Published:** 2010-12-10

**Authors:** Elizabeth V Cyarto, Kay L Cox, Osvaldo P Almeida, Leon Flicker, David Ames, Gerard Byrne, Keith D Hill, Christopher D Beer, Dina LoGiudice, Kana Appadurai, Muireann Irish, Emma Renehan, Nicola T Lautenschlager

**Affiliations:** 1 National Ageing Research Institute, Melbourne, Australia; 2 School of Medicine and Pharmacology, University of Western Australia, Perth, Australia; 3 School of Psychiatry & Clinical Neurosciences, University of Western Australia, Perth, Australia; 4 Western Australian Centre for Health & Ageing, Centre for Medical Research, University of Western Australia, Perth, Australia; 5 Department of Psychiatry, Royal Perth Hospital, Perth, Australia; 6 Department of Geriatric Medicine, Royal Perth Hospital, Perth, Australia; 7 Academic Unit for Psychiatry of Old Age, St. Vincent's Health, Department of Psychiatry, University of Melbourne, Melbourne, Australia; 8 Melbourne Health, Melbourne, Australia; 9 School of Medicine, University of Queensland, Brisbane, Australia; 10 Older Persons' Mental Health Service, Royal Brisbane and Women's Hospital, Brisbane, Australia; 11 Faculty of Health Sciences, La Trobe University and Northern Health, Melbourne, Australia; 12 Department of Internal Medicine and Aged Care, Royal Brisbane and Women's Hospital, Brisbane, Australia; 13 Neuroscience Research Australia, Sydney, Australia

## Abstract

**Background:**

Observational studies have documented a potential protective effect of physical exercise in older adults who are at risk for developing Alzheimer's disease. The Fitness for the Ageing Brain II (FABS II) study is a multicentre randomized controlled clinical trial (RCT) aiming to determine whether physical activity reduces the rate of cognitive decline among individuals with Alzheimer's disease. This paper describes the background, objectives of the study, and an overview of the protocol including design, organization and data collection methods.

**Methods/Design:**

The study will recruit 230 community-dwelling participants diagnosed with Alzheimer's disease. Participants will be randomly allocated to two treatment groups: usual care group or 24-week home-based program consisting of 150 minutes per week of tailored moderate physical activity. The primary outcome measure of the study is cognitive decline as measured by the change from baseline in the total score on the Alzheimer's disease Assessment Scale-Cognitive section. Secondary outcomes of interest include behavioral and psychological symptoms, quality of life, functional level, carer burden and physical function (strength, balance, endurance, physical activity). Primary endpoints will be measured at six and twelve months following the baseline assessment.

**Discussion:**

This RCT will contribute evidence regarding the potential benefits of a systematic program of physical activity as an affordable and safe intervention for people with Alzheimer's disease. Further, if successful, physical activity in combination with usual care has the potential to alleviate the symptoms of Alzheimer's disease and improve its management and the quality of life of patients and their carers.

**Trial Registration:**

Australia New Zealand Clinical Trials Registry ACTRN12609000755235

## Background

Advancing age increases the risk of dementia [1], of which Alzheimer's disease (AD) is the most common cause. Available projections indicate that the number of people with dementia worldwide will increase from 35.6 million in 2010 to 115.4 million people by 2050 [2], with dementia expected to become the major social and economic health challenge of the 21^st ^century.

Alzheimer's disease is characterized by a progressive deterioration in memory and other higher cortical functions that ultimately leads to a loss of independent living skills [3]. Despite the availability of palliative medication with cholinesterase inhibitors and memantine [4], there is currently no cure for AD. There is growing evidence that some environmental factors such as physical and cognitive activity [5,6], decrease the risk of AD [7] and improve the behaviour of patients [8], but it is unclear if such lifestyle interventions can also delay the progression of cognitive decline once the diagnosis of AD has been established [9].

Prospective cohort studies indicate that PA is associated with reduced incidence of dementia [10]. Middleton et al. [11] reported that women who were physically active across the life course had a lower prevalence of cognitive impairment in later life. The association between PA and cognitive function was evident even when exercise is limited to later life [12]. However, as Leone et al. [13] noted, these findings are limited by methodological issues such as survivorship bias and confounding, the latter arising because the exposure to PA is not random in observational studies.

A recent randomised trial (RCT) showed that six months of PA decreased the rate of cognitive decline in older people with subjective memory complaints or Mild Cognitive Impairment (MCI) [14], a group that is at increased risk of developing AD [15]. Subsequently, Baker and colleagues [16] found that six months of high-intensity aerobic activity (75-85% of heart rate reserve) improved executive control processes in sedentary women with MCI. Possible mechanisms mediating the cognitive enhancing effect of PA include alterations of cerebral vascular functioning and brain perfusion, environment enrichment and stimulation of synaptogenesis [14]. A Cochrane review of PA trials for individuals with MCI is currently under way [17].

Few RCTs of PA in AD have been published to date. Most evidence in this area comes from studies conducted with nursing home residents or participants with moderate to severe AD, and the primary outcomes of interest tend to be functional status and/or mood [8,18-20]. The intervention provided in the largest trial, with 134 participants from five nursing homes, was a group-based multi-component exercise program [18]. After 12 months, participants in the exercise group experienced a slower decline in their performance of activities of daily living than those in the control group, but there was no change in behavioural or psychological symptoms (BPSD). In a pilot RCT, Steinberg et al. [19] examined the effect of a home-based, carer-supervised program of walking, strength training and balance exercises on cognition, secondary to measures of functional performance, in 27 people with AD. Although the small sample size likely precluded finding any significant differences between groups in cognitive functioning, the investigators found that the exercise program was safe, could be supervised by caregivers and had good compliance.

The objectives of the present study are to conduct a methodologically rigorous RCT investigating the potential benefits of PA on cognition, well-being, function and BPSD in community-dwelling participants diagnosed with mild to moderate AD. We will also examine whether the intervention eases stress and burden of care, an area that is often neglected in such RCTs [21]. This paper describes the design of the Fitness for the Ageing Brain Study II (FABS II).

## Methods/Design

### Study Design

**Figure 1:**
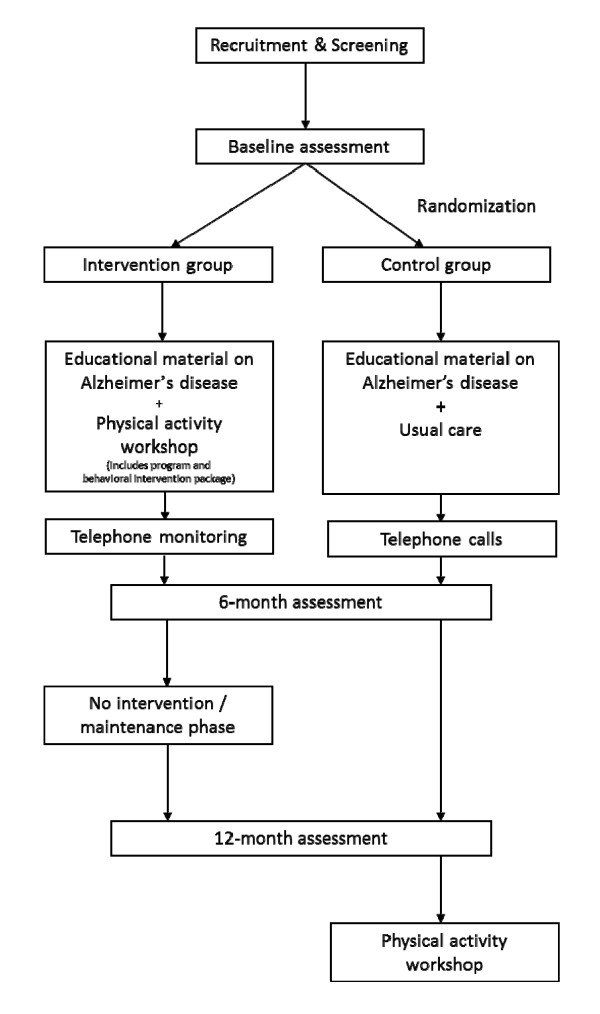
**Study Design**.

FABS II is a single blind RCT (Figure 1). The CONSORT statement has been used as a framework for development of the methodology for this project.

### Participants

230 community-dwelling older adults diagnosed with AD will be recruited across three sites in Australia (Melbourne, Perth and Brisbane). Participants will be included in the study if they satisfy the following criteria: (i) diagnosed with probable or possible AD according to NINCDS-ADRDA criteria [3], (ii) score of 10 or greater on the Standardised Mini Mental State Examination (SMMSE) [22], (iii) community dwelling, (iv) no clinically significant depression, (v) understands written and spoken English, (vi) contact with a friend or family member (carer) for at least 10 hours per week, who is also willing to participate in the trial, and (vii) no other major neurological history or medical condition that contraindicates PA.

Participants will be excluded if they have limited mobility (e.g. unable to walk alone or require a walking aid for balance), show evidence of clinically significant aphasia or pervasive depression, current history of alcohol dependence, have an unstable or life-threatening medical condition, or are participating in another RCT.

Diagnosis of AD will be confirmed through medical records obtained from the participant's treating general practitioner and specialist. These records will be reviewed by members of the research team who are either experienced Geriatricians or Old Age Psychiatrists to confirm the participant's suitability for inclusion.

This study is funded by the National Health and Medical Research Council of Australia. Ethics approval has been obtained from the Human Research Ethics Committee(s) of each participating site and the project complies with the Declaration of Helsinki.

### Recruitment and Screening

Potential participants and their carers will be identified and recruited via local physicians, Memory Clinics and specialists. Additionally, flyers advertising the study and media activities will invite suitable older adults living in the community to consider participation. The research team will contact the participant and next of kin by telephone to check the suitability of the participant according to the study inclusion and exclusion criteria using a strict screening protocol. The 15-item Geriatric Depression Scale (GDS - 15) [23] is included as part of the phone screening to establish the presence of clinically relevant symptoms of depression, excluding potential participants with a score of 6 and higher. The name and contact details for the participant's GP and specialist will be obtained, and the participant will be asked to sign and return a release of medical information form to allow the study team to access their medical records. The participant's GP will also be asked to consent to their patient's involvement in the study. This is another way of determining the suitability of FABS II for the potential participant and ensuring his/her safety during the course of the trial.

### Assessments

Participants will be assessed at baseline and after six months and twelve months in the study (see Table 1 for an overview). There will be two components to the assessments comprising cognitive measures and physical function measures/physical activity questionnaires. Unless stated otherwise, all measures will be administered at each time point. The research assistant (RA) administering the cognitive assessment (Cognitive RA) will be trained by either a Geriatrician or an Old Age Psychiatrist at each site. To ensure that the Cognitive RA remains blind to study group allocation, the physical assessments will be conducted by a separate PA RA. The PA RA will collect demographic and health information via participant interview. The PA RA at each site will be trained by the same chief investigator with a background in exercise physiology to ensure standardization of procedures across all three sites.

**Table 1:** Outline of the assessments and timelines of the FABS II.

*Assessment Tool*	Telephone Screen	Baseline (0 weeks)	Post-Intervention (24 weeks)	12 months (52 weeks)
Geriatric Depression Scale - 15 item	X	X	X	X

Cambridge Contextual Reading Test		X		

Alzheimer's disease Assessment Scale - Cognitive section		X	X	X

Standardized Mini-Mental State Examination		X	X	X

Clinical Dementia Rating Scale		X	X	X

Quality of Life - AD		X	X	X

Neuropsychiatric Inventory		X	X	X

Instrumental Activities of Daily Living		X	X	X

Activities of Daily Living		X	X	X

Short Form-36 version 2 (SF-36v2)		X	X	X

Zarit Burden Interview		X	X	X

Resting blood pressure		X	X	X

Height, weight, body composition, girths		X	X	X

Step test		X	X	X

Sit-to-stand test		X	X	X

Grip strength		X	X	X

Timed Up and Go test		X	X	X

Two-minute walk		X	X	X

Community Healthy Activities Model Program for Seniors (CHAMPS) questionnaire		X	X	X

Stages of Change		X	X	X

Satisfaction with Life		X	X	X

Self-Efficacy Questionnaire		X	X	X

DNA sample collection		X		

The × indicates at which point of the trial the respective assessments took place. Follow-up times relate to baseline testing.

#### Measures administered by the Cognitive RA

The total score on the Alzheimer's disease Assessment Scale - Cognitive section (ADAS-cog) [24] will be the primary outcome measure of the study. The ADAS-cog consists of an 11-item battery of short neuropsychological tests and is widely used to monitor the progression of cognitive deficits in clinical trials with patients with AD. Here we will use a version incorporating a delayed verbal recall task. Higher scores indicate increased severity of cognitive impairment (maximum score is 85).

The following nine assessments comprise the secondary outcome measures. The Cambridge Contextual Reading Test (CCRT) [25] includes the words of the National Adult Reading Test (NART), embedding them within appropriate semantic and syntactic contexts to provide a reliable measure of pre-morbid intelligence. The CCRT is only administered at baseline.

The SMMSE [22] is a modified version of the traditional MMSE [26], which benefits from greater objectivity through more specific scoring examples as well as alternatives for repeated testing of the registration and delayed recall items. A score of less than 10 on the SMMSE will be used to exclude participants, as these individuals would most likely be too cognitively impaired to follow the intervention instructions.

The Clinical Dementia Rating Scale (CDR) [27] is a global rating scale for dementia incorporating information from the primary carer across the domains of memory, orientation, judgment and problem-solving, community affairs, home and hobbies, and personal care. The subscores on each domain are ranked according to severity and an algorithm is used to compute the total score.

The Quality of Life in AD (QoL-AD) [28] is a 13-item questionnaire which requires the participant and carer to independently rate the participant's quality of life across a number of domains on a scale ranging from poor (1 point) to excellent (4 points).

The Neuropsychiatric Inventory (NPI) [29] is a well-established assessment of 12 different BPSD common in AD including delusions, hallucinations, agitation, depression, anxiety, euphoria, apathy, disinhibition, irritability, aberrant motor behaviour, night-time behaviour, appetite and eating disorders. The carer rates each symptom according to the frequency and severity as well as the degree of distress the symptom causes them.

The Instrumental Activities of Daily Living scale (IADL) [30] is widely used to assess independent living skills and is useful in identifying improvement or deterioration over time. The carer rates the AD participant across eight functional abilities, including use of the telephone, shopping, food preparation, housekeeping, laundry, mode of transport, taking medications and handling finances.

The Index of Independence in Activities of Daily Living scale (ADL) [31] is a six-item questionnaire where the carer is required to rate personal and domestic ADLs such as bathing, dressing, toileting, transfers, continence, and feeding. The number of activities in which the participant is deemed 'dependent' is counted.

The Short Form-36v2 [32] is a 36-item questionnaire that assesses the health and well-being of the carer across eight dimensions; physical functioning, social functioning, role limitations due to physical problems, role limitations due to emotional problems, mental health, energy/vitality, pain, and general health perception. Health change over the past year is also assessed. For each dimension of the SF36v2, item scores are coded, summed and transformed on a scale from 0 (worst possible health state measured by questionnaire) to 100 (best possible health state).

The Zarit Burden Interview (ZBI) [33] is a 22-item self-rating scale that examines the degree of burden experienced by the carer associated with functional and behavioural impairments of the person with AD. The items are phrased subjectively and focus on the affective response of the carer.

#### Measures administered by the PA RA

Resting blood pressure will be assessed after a five-minute resting period, with five measurements taken at two-minute intervals with a digital blood pressure monitor (model UA-767PC, A&D Co., Ltd., Saitama, Japan).

Height, body weight, body composition and girths will be measured in light clothing and bare feet. Height will be measured using a fixed stadiometer. Body weight and composition (fat mass, fat free mass) will be assessed using a bio-impedance analyzer, following the procedures provided by the manufacturer (model TBF-300, Tanita Corporation of America, Inc., Illinois, USA). Waist and hip girths will be measured three times (median score used) using a retractable steel tape measure (Lufkin W606PM Cooper industries SC, USA) [34].

A step test using a 7.5 cm square wooden block will be used to assess dynamic balance [35]. The participant places one foot on and off the block as many times as possible in 15 seconds without using hand support. Performance for each leg stepping is assessed separately, and the lowest score will be recorded. The Timed Up and Go (TUG) test will be used to assess mobility [36]. Participants will be seated in a chair with arms resting on the arm rests and asked to get up and walk three meters as fast and safely as they can, turn around and return to a seated position. Functional lower limb strength will be measured using the sit-to-stand test (five chair stands) [37], with participants instructed to stand up and sit down as quickly as possible with or without use of their arms. Maximum voluntary hand grip strength (both hands) will be assessed using a Smedleys dynamometer [34]. The participant will stand against a wall. Holding the dynamometer vertically above the head, the participant will be instructed to squeeze as hard as possible while bringing the arm back down to their side. A 2-minute walk around a 20-metre measured course will be utilized to assess cardiovascular endurance [38]. Participants will be instructed to walk as fast as possible around the course as many times as they can in two minutes. Participants will be allowed to stop walking during the testing period if they needed to, and any rests will be included in the total 2-minute test period. The maximum distance walked, heart rate and rate of perceived exertion [39] will be recorded.

Physical activity will be assessed using the Community Healthy Activities Model Program for Seniors (CHAMPS) physical activity questionnaire [40]. Assessment will be made of the frequency, minutes, and calorie expenditure per week of all, low, moderate, high and very high intensity activities.

Participants will be provided with a pedometer (Digi-Walker SW-200, Yamax Inc., Tokyo, Japan) and asked to wear it for five weekdays and the weekend, following the baseline visit, to objectively measure their weekly PA. The pedometer will be put on immediately upon wakening and taken off just before retiring at night. It will also be removed whilst bathing or showering and the time off recorded on the pedometer diary. Participants and carers will be shown how to wear and use the pedometer and how to complete the diary. Participants will be instructed to maintain their usual activity pattern during the monitoring period.

The stage of PA behavior will be assessed using the Stage of Change Instrument (SCI), a five-item questionnaire asking participants to indicate their current level of activity [41]. The frequency and minutes per week of regular moderate intensity PA will also be measured using this questionnaire.

Exercise self-efficacy will be assessed with the five-item Self-Efficacy Questionnaire (SEQ) asking participants how confident they were to exercise in certain adverse conditions with responses on a 5-point scale ranging from "not at all sure" to "very sure" [42].

Satisfaction with Life will be measured using a five-item questionnaire with responses on a seven-point scale ranging from "strongly disagree" to "strongly agree" [43].

### DNA Sample Collection

APOE genotyping will be performed at baseline testing to adjust for APOE genotype in the final analysis and to investigate whether there is an interaction between APOE genotype and the benefits of PA influencing the primary and secondary outcomes. Participants will be asked to provide a 2 ml saliva sample using a DNA self-collection kit (Oragene DNA OG-250 Disc Format, DNA Genotek Inc, Ontario, Canada). They will be stored at -80°C at the Department of Clinical Pathology and Biochemistry at the Royal Perth Hospital. Samples from Brisbane and Melbourne will be mailed to Perth. All samples will be batched and processed once the final baseline assessment has been completed.

### Randomization and Blinding

Random allocation to intervention or control group will be performed using Stata and the user-written program ralloc [44]. Allocation numbers will be drawn by an investigator not directly involved in the recruitment or assessment of participants. Each of the three study sites will be provided with sealed opaque envelopes numbered 1-100 and used in that order. After a participant completes his/her baseline assessment, the PA RA will open the next consecutive envelope. Inside the envelope there will either be the letter A (physical activity group) or B (control group). Due to logistic difficulties, participants will not be blind to the intervention and sham PA will not be used. However, the "clinical staff" involved in the collection of endpoints will not be aware of group allocation (single blind). Blinding will be supported by the performance of cognitive and physical assessments at different locations and explicit instructions to participants and clinical research staff not to discuss issues related to PA during the assessments. The PA RA will inform participants of their group allocation by telephone and will instruct them not to share that information with research staff involved in their future cognitive assessments.

### Intervention

The intervention period will be 24 weeks. The intervention will comprise three components: the PA program, the behavioral intervention package, and telephone monitoring. Participants randomized to the intervention will return with their carer ("coach") for a PA workshop within two to four weeks of their baseline visit. During this 60-minute session, the PA RA will give participants their program manual and explain the participants' PA program and the behavioral intervention package.

#### Physical activity program

Participants will be advised to perform at least 150 min/week of moderate PA as per the new PA recommendations for older people [45]. Where walking is a suitable and acceptable option to the participant and coach, this will be a primary PA recommendation. Examples will be given on how this can be achieved. The program will be individualized based on the CHAMPS and SCI data, and the person's interests. Activities prescribed will take into account health problems or other limitations, and participants will be instructed to start slowly and progress gradually taking eight weeks to reach the target amount and intensity. Participants will be encouraged to achieve the 150 min/week by completing 3 × 50 minutes sessions (most PA classes range between 45-60 minutes). In previous studies with middle-aged and older women using this format, we have demonstrated good retention, adherence and improvements in cardiovascular fitness and blood pressure [46]. Participants who already perform 150 min/week of PA will be encouraged to increase their PA by adding one 50-minute session. To reduce the burden on coaches, some participants might choose to complete activities in community centers. The coach will be invited to perform physical activities alongside participants, if appropriate.

In order to maintain standardization of the physical activity programs one CI will monitor the programs prescribed by the RA at each site. Further, to maintain quality control another CI with a PA background will review programs from a random selection on a regular basis from all sites.

The PA program will include instructions on how to read the program, complete the activities, record their sessions, and exercise safely. Participants will be given a simple diary and the coach will be asked to verify the activity record the PA in the diary. The diaries, once completed, will be retained by the participant and returned at their six-month visit.

After the first 24 weeks, participants will be asked to continue with their PA program for a further 24 weeks however, there will be no further monitoring or contact between the researchers and participants except for three newsletters. At the end of 12 months, intervention participants will be asked to complete a brief questionnaire regarding their program adherence for the previous six months.

Adherence during the six month active intervention will be calculated from the number of sessions completed and recorded on the exercise diaries. This will be expressed as a percentage of the number of sessions completed relative to the number of sessions prescribed.

#### Behavioral Intervention

Participants and coaches will receive the same educational material about AD as the control group. The intervention program will be based on the Stages of Change model modified for PA [41], which has been shown to be effective in increasing and maintaining PA in middle-aged and older women [46]. This model proposes that individuals move through five stages when they adopt a new behavior. From participants' SCI data, a behavioral program will be developed to assist them to move to the next stage of PA. Strategies to increase adherence to the program will be discussed during the workshop and worksheets will be included in a manual. Topics will include tips on how to exercise, benefits and costs of exercising, rewards of exercise, goal setting, time management, etc.

During the 24-week trial, the PA RA will mail newsletters at regular intervals to participants, which contain additional motivational information. This will also be reinforced during the five telephone calls made at regular intervals. Participants and coaches will be contacted by telephone for a standardized and structured 15- minute interview to monitor and give feedback on their progress and encourage their continuing adherence.

Participants will also be given a report after their follow-up assessments. Giving feedback about progress and increasing participants' perceived benefits of being more physically active has been shown to increase program adherence [47].

#### Content and Program Evaluation

At the end of the intervention period, the intervention group participants and the coach will be asked to complete a brief interview questionnaire on the content and processes of the program including how easy the program was to understand and follow, and any barriers that were encountered.

### Control Group

Control group (usual care) participants and carers will receive educational material about AD and recommendations for a healthy lifestyle (other than PA). Participants will be contacted by telephone at the same frequency as the intervention group to ensure that the control and intervention group have similar treatment except for the actual intervention. Conversation for this group will be limited to their general health and will not include discussion about physical activity. These activities, together with the benefit of follow-up assessments will strengthen the adherence or ongoing participation of the usual care group. At the conclusion of the study, these participants will be offered the opportunity to attend a session on PA.

### Statistical Methods

Participants who drop out during the trial will be invited to return for the follow-up assessments. We will use imputation by chain equations (ICE) to estimate possible missing outcomes in an intention-to-treat analysis (primary analysis). We will use multilevel regression models to take into account repeated measures and intra-individual variability (mixed models). These models will be adjusted for confounding should the randomization produce unbalanced groups.

#### Sample Size and Power Calculation

In a pilot study conducted by this research team, the ADAS-Cog score of participants in the control group (usual care, n = 10) increased 4.47 ± 6.36 points in six months (indicating cognitive decline) whereas the patients in the intervention group (physical activity, n = 12) increased 2.21 ± 4.88 points. Based on this pilot data, recruiting 115 participants in each of the two groups (total = 230) at baseline will result in a power of 80% to detect a difference of 2.2 points between the groups (alpha = 0.05, 2-tailed). This sample size also allows for an estimated 15% drop out rate at follow-up.

## Discussion

Few RCTs have tested the effect of physical activity in community dwelling people with AD, and none have assessed the beneficial effects of physical activity in terms of family caregiver burden and wellbeing [21]. By building on the success of the FABS I trial involving people with MCI [14], the FABS II study attempts to address this gap in the literature. The study will also evaluate the effectiveness of the strategies used to promote short-term and longer-term adherence to physical activity in this population. The findings have the potential to inform practitioners about successful strategies and provide the impetus for translation into community programs. Further, the trial not only focuses on the participant's global cognitive and clinical symptoms, functional level and quality of life but also on the indirect positive effects that may be experienced by the caregiver. From the 12-month follow-up assessment, we also hope to provide evidence regarding the continuation of such benefits.

Given the projected rising global incidence of AD over the next several decades, the FABS II trial is timely. If the current study protocol should prove successful, this intervention will represent an affordable and safe method, in combination with standard pharmacological treatments, to alleviate the symptoms of AD. We anticipate that the results of this study will contribute to the overall understanding and management of cognitive and behavioural symptoms of AD as well as potentially improving the quality of life of patients and their carers.

## Competing interests

The authors declare that they have no competing interests.

## Authors' contributions

All authors are members of the FABS II research team and participated in the implementation of the study. The physical activity and behavioral intervention program was developed by KC. EC is the project coordinator and drafted the manuscript. NL, KC, OA, LF, DA, GB, KH, CB, DL and KA conceived of the study, participated in its design and coordination and critically reviewed the manuscript. MI helped to draft the manuscript and contributed to its critical review. ER contributed to critical review of the manuscript. All authors read and approved the final manuscript.

## References

[B1] Jorm AF Copeland JRM, Abou-Saleh MT, Blazer DG Dementia epidemiology: prevalence and incidence Principles and practice of geriatric psychiatry 2002 2 Chichester: Wiley 195 197 full_text

[B2] Alzheimer's Disease International World Alzheimer Report 2009 2009 London: Alzheimer's Disease International

[B3] McKhann G Drachman D Folstein MF Katzman R Price D Stadlan EM Clinical diagnosis of Alzheimer's disease: report of the NINCDS-ADRDA Work Group under the auspices of Department of Health and Human Services Task Force on Alzheimer's Disease Neurology 1984 34 7 939 944 6610841 10.1212/wnl.34.7.939

[B4] Rabins PV Lyketsos CG Cholinesterase inhibitors and memantine have a role in the treatment of Alzheimer's disease Nat Clin Pract Neurol 2006 2 11 578 579 10.1038/ncpneuro0269 17057739

[B5] Middleton LE Yaffe K Promising strategies for the prevention of dementia Arch Neurol 2009 66 10 1210 1215 10.1001/archneurol.2009.201 19822776 PMC2762111

[B6] Scarmeas N Luchsinger JA Schupf N Brickman AM Cosentino S Tang MX Stern Y Physical activity, diet, and risk of Alzheimer disease JAMA 2009 302 6 627 637 10.1001/jama.2009.1144 19671904 PMC2765045

[B7] Larson EB Wang I Bowen JD McCormick WC Teri L Crane P Kukull WA Exercise is associated with reduced risk for incident dementia among persons 65 years of age and older Ann Intern Med 2006 144 73 81 16418406 10.7326/0003-4819-144-2-200601170-00004

[B8] Teri L Gibbons LE McCurry SM Logsdon LG Buchner DM Barlow WE Kukull WA LaCroix AZ McCormick W Larson EB Exercise plus behavioral management in patients with Alzheimer disease JAMA 2003 290 15 2015 2022 10.1001/jama.290.15.2015 14559955

[B9] Weuve J Kang JH Manson JE Breteler MMB Ware JH Grodstein F Physical activity, including walking, and cognitive function in older women JAM A 2004 292 1454 1461 10.1001/jama.292.12.1454 15383516

[B10] Podewils LJ Guallar E Kuller LH Fried LP Lopez OL Carlson M Lyketsos CG Physical activity, APOE genotype, and dementia risk: findings from the Cardiovascular Health Cognition Study Am J Epidemiol 2005 161 639 651 10.1093/aje/kwi092 15781953

[B11] Middleton LE Barnes DE Lui LY Yaffe K Physical activity over the life course and its association with cognitive performance and impairment in old age J Am Geriatr Soc 2010 58 7 1322 1326 10.1111/j.1532-5415.2010.02903.x 20609030 PMC3662219

[B12] van Gelder BM Tijhuis MAR Kalmijn S Giampaoli S Nissinen A Kromhout D Physical activity in relation to cognitive decline in elderly men: The FINE Study Neurology 2004 63 2316 2321 15623693 10.1212/01.wnl.0000147474.29994.35

[B13] Leone E Deudon A Robert P Physical activity, dementia, and BPSD J Nutr Health Aging 2008 12 7 457 460 10.1007/BF02982706 18615227 PMC12892704

[B14] Lautenschlager NT Cox KL Flicker L Foster JK van Bockxmeer FM Xiao J Greenop KR Almeida OP Effect of physical activity on cognitive function in older adults at risk for Alzheimer disease JAMA 2008 300 9 1027 1037 10.1001/jama.300.9.1027 18768414

[B15] Petersen RC Mild cognitive impairment as a diagnostic entity J Intern Med 2004 256 3 183 194 10.1111/j.1365-2796.2004.01388.x 15324362

[B16] Baker LD Frank LL Foster-Schubert K Green PS Wilkinson CW McTiernan A Plymate SR Fishel MA Watson GS Cholerton BA Effects of aerobic exercise on mild cognitive impairment: A controlled trial Arch Neurol 2010 67 1 71 79 10.1001/archneurol.2009.307 20065132 PMC3056436

[B17] Orgeta V Regan C Orrell M Physical activity for improving cognition in older people with mild cognitive impairment (Protocol) Cochrane Database Syst Rev 2010 1 CD008198

[B18] Rolland Y Pillard F Klapouszczak A Reynish E Thomas D Andrieu S Riviere D Vellas B Exercise program for nursing home residents with Alzheimer's disease: A 1-year randomized, controlled trial J Am Geriatr Soc 2007 55 2 158 165 10.1111/j.1532-5415.2007.01035.x 17302650

[B19] Steinberg M Sheppard Leoutsakos J-M Podewils LJ Lyketsos CG Evaluation of a home-based exercise program in the treatment of Alzheimer's disease: The Maximizing Independence in Dementia (MIND) study Int J Geriatr Psychiatry 2009 24 680 685 10.1002/gps.2175 19089875 PMC5172460

[B20] Williams CL Tappen RM Effect of exercise on mood in nursing home residents with Alzheimer's disease Am J Alzheimers Dis Other Demen 2007 22 389 397 10.1177/1533317507305588 17959874 PMC2134914

[B21] Forbes D Forbes S Morgan DG Markle-Reid M Wood J Culum I Physical activity programs for persons with dementia Cochrane Database Syst Rev 2008 3 CD006489 18646158 10.1002/14651858.CD006489.pub2

[B22] Molloy DW Alemayehu E Roberts R Reliability of a Standardized Mini-Mental State Examination compared with the traditional Mini-Mental State Examination Am J Psychiatry 1991 148 1 102 105 1984692 10.1176/ajp.148.1.102

[B23] Almeida OP Almeida SA Short versions of the Geriatric Depression Scale: a study of their validity for the diagnosis of a major depressive episode according to ICD-10 and DSM-IV Int J Geriatr Psychiatry 1999 14 858 865 10.1002/(SICI)1099-1166(199910)14:10<858::AID-GPS35>3.0.CO;2-8 10521885

[B24] Rosen WG Mohs RC Davis KL A new rating scale for Alzheimer's Disease Am J Psychiatry 1984 11 1356 1364 10.1176/ajp.141.11.1356 6496779

[B25] Beardsall L Huppert FA Improvement in NART word reading in demented and normal older persons using the Cambridge Contextual Reading Test J Clin Exper Neuropsych 1994 16 2 232 242 10.1080/01688639408402634 8021310

[B26] Folstein MF Folstein SE McHugh PR 'Mini-mental state': a practical method for grading the cognitive state of patients for the clinician J Psychiatr Res 1975 12 3 189 198 10.1016/0022-3956(75)90026-6 1202204

[B27] Morris J The CDR: Current version and scoring rules Neurology 1993 43 11 2412 2413 8232972 10.1212/wnl.43.11.2412-a

[B28] Logsdon RG Gibbons LE McCurry SM Teri L Quality of life in Alzheimer's disease: patient and carer reports J Mental Health Aging 1999 5 1 21 32

[B29] Cummings JL Mega M Gray K Rosenberg-Thompson S Carusi DA Gornbein J The Neuropsychiatric Inventory: comprehensive assessment of psychopathology in dementia Neurology 1994 44 2308 2314 7991117 10.1212/wnl.44.12.2308

[B30] Lawton MF Brody EM Assessment of older people: self-maintaining and instrumental activities of daily living Gerontologist 1969 9 179 186 5349366

[B31] Katz S Ford AB Moskowitz RW Jackson BA Jaffe MW Studies of illness in the aged. The Index of ADL: A standardized measure of biological and physical function JAMA 1963 185 914 919 14044222 10.1001/jama.1963.03060120024016

[B32] Hawthorne G Osborne RH Taylor A Sansoni J The SF36 Version 2: critical analyses of population weights, scoring algorithms and population norms Qual Life Res 2007 16 4 661 673 10.1007/s11136-006-9154-4 17268926

[B33] Zarit SH Reever KE Bach-Peterson J Relatives of the impaired elderly: Correlates of feelings of burden Gerontologist 1980 20 649 655 7203086 10.1093/geront/20.6.649

[B34] Gore CJ Edwards DA Australian fitness norms: a manual for fitness assessors 1992 The Health Development Foundation

[B35] Hill KD Bernhardt J McGann AM Maltese D Berkovits D A new test of dynamic standing balance for stroke patients: Reliability, validity, and comparison with healthy elderly Physiother Can 1996 48 257 262 10.3138/ptc.48.4.257

[B36] Podsiadlo D Richardson S The timed "up and go": A test of basic functional mobility for frail elderly persons J Am Geriatr Soc 1991 39 142 148 1991946 10.1111/j.1532-5415.1991.tb01616.x

[B37] McCarthy EK Horvat MA Holtsberg PA Wisenbaker JM Repeated chair stands as a measure of lower limb strength in sexagenarian women J Gerontol A Biol Sci Med Sci 2004 59 11 1207 1212 15602077 10.1093/gerona/59.11.1207

[B38] Brooks D Parsons J Tran D Jeng B Gorczyca B Newton J Lo V Dear C Silaj E Hawn T The Two-minute Walk Test as a measure of functional capacity in cardiac surgery patients Arch Phys Med Rehabil 2004 85 1525 1530 10.1016/j.apmr.2004.01.023 15375829

[B39] Borg GAV Psychological basis of physical exertion Med Sci Sports Exerc 1982 14 377 381 7154893

[B40] Stewart AL Mills KM King AC Haskell WL Gillis D Ritter PL CHAMPS physical activity questionnaire for older adults: outcomes for interventions Med Sci Sports Exerc 2001 33 7 1126 1141 11445760 10.1097/00005768-200107000-00010

[B41] Marcus BH Banspach SW Lefebvre RC Rossi JS Carleton RA Abrams DB Using the stages of change model to increase the adoption of physical activity among community participants Am J Health Promotion 1992 6 424 429 10.4278/0890-1171-6.6.424 10146803

[B42] Marcus BH Selby VC Niaura RS Rossi JS Self-efficacy and the stages of exercise behaviour change Res Q Exerc Sport 1992 63 60 66 1574662 10.1080/02701367.1992.10607557

[B43] Pavot W Diener E Colvin CR Sandvik E Further validation of the satisfaction with life scale: Evidence for the cross-method convergence well-being measures J Pers Assess 1991 57 1 149 161 10.1207/s15327752jpa5701_17 1920028

[B44] Ryan P sxd1: Random allocation of treatments in blocks Stata Technical Bulletin 1998 41 43 46

[B45] Sims J Hill K Hunt S Haralambous B Physical activity recommendations for older Australians Australas J Ageing 2010 29 2 81 87 10.1111/j.1741-6612.2009.00388.x 20553539

[B46] Cox KL Burke V Gorely TJ Beilin LJ Puddey IB Controlled comparison of retention an adherence in home- vs center-initiated exercise interventions in women ages 40-65 years: The S.W.E.A.T. Study (Sedentary Women Exercise Adherence Trial) Prev Med 2003 36 17 29 10.1006/pmed.2002.1134 12473421

[B47] Rothman AJ Toward a theory-based analysis of behavioural maintenance Health Psychol 2000 1 Suppl 664 669 10.1037/0278-6133.19.suppl1.64 10709949

